# YOLO-ACE: Enhancing YOLO with Augmented Contextual Efficiency for Precision Cotton Weed Detection

**DOI:** 10.3390/s25051635

**Published:** 2025-03-06

**Authors:** Qi Zhou, Huicheng Li, Zhiling Cai, Yiwen Zhong, Fenglin Zhong, Xiaoyu Lin, Lijin Wang

**Affiliations:** 1 College of Computer and Information Sciences, Fujian Agriculture and Forestry University, Fuzhou 350002, China; 5221139009@fafu.edu.cn (Q.Z.); 52411049022@fafu.edu.cn (H.L.); zhilingcai@126.com (Z.C.); yiwzhong@fafu.edu.cn (Y.Z.); lijinwang@fafu.edu.cn (L.W.); 2 Key Laboratory of Smart Agriculture and Forestry, Fujian Province University, Fuzhou 350002, China; 3 College of Horticulture, Fujian Agriculture and Forestry University, Fuzhou 350002, China; zhong591@fafu.edu.cn; 4 Fujian Key Laboratory of Big Data Application and Intellectualization for Tea Industry, Wuyi University, Wuyishan 354300, China

**Keywords:** weed detection, deep learning, YOLOv5s, attention mechanism

## Abstract

Effective weed management is essential for protecting crop yields in cotton production, yet conventional deep learning approaches often falter in detecting small or occluded weeds and can be restricted by large parameter counts. To tackle these challenges, we propose YOLO-ACE, an advanced extension of YOLOv5s, which was selected for its optimal balance of accuracy and speed, making it well suited for agricultural applications. YOLO-ACE integrates a Context Augmentation Module (CAM) and Selective Kernel Attention (SKAttention) to capture multi-scale features and dynamically adjust the receptive field, while a decoupled detection head separates classification from bounding box regression, enhancing overall efficiency. Experiments on the CottonWeedDet12 (CWD12) dataset show that YOLO-ACE achieves notable mAP@0.5 and mAP@0.5:0.95 scores—95.3% and 89.5%, respectively—surpassing previous benchmarks. Additionally, we tested the model’s transferability and generalization across different crops and environments using the CropWeed dataset, where it achieved a competitive mAP@0.5 of 84.3%, further showcasing its robust ability to adapt to diverse conditions. These results confirm that YOLO-ACE combines precise detection with parameter efficiency, meeting the exacting demands of modern cotton weed management.

## 1. Introduction

Weeds, diseases, and pests persist as major threats to crop health and yield [1], particularly in cotton cultivation. Weed intrusion can significantly reduce annual cotton yields, presenting severe challenges to agricultural production [2]. Weeds not only compete with crops for vital resources such as soil nutrients, water, and sunlight, but, if neglected, can significantly decrease cotton production and heighten the threat of other biological pests [3]. Historically, farmers have depended on personal experience or expert guidance to address these issues, yet such approaches can be subjective and time-consuming, often resulting in pesticide overuse [4]. Consequently, modern technologies—such as precision spraying methods leveraging machine vision and artificial intelligence [5] and disease diagnosis techniques [6]—are crucial to advancing agricultural sustainability. These technologies reduce pesticide usage and environmental impact while improving crop yield and quality, which is especially significant in the cotton industry, a cornerstone of the global economy.

Initially, weed and pest identification relied heavily on traditional machine learning techniques [7,8,9,10], effective in image classification but burdened by manual feature extraction and integration. Such approaches are labor-intensive and prone to subjective bias, making them less suitable for the varied and complex conditions found in cotton fields. With the rise of Convolutional Neural Networks (CNNs), however, deep learning has enabled major strides in crop classification, weed detection, and yield prediction [11]. CNN-based solutions operate without handcrafted features and allow end-to-end training, offering faster and more accurate detection than traditional methods, which remain restricted by both feature engineering and processing speed.

Within deep learning, two-stage models like the RCNN series excel in accuracy but incur higher computational costs. Single-stage models, such as You Only Look Once (YOLO) [12] and Single Shot MultiBox Detector (SSD) [13], prioritize rapid detection and maintain competitive precision—attributes especially pertinent for in-field weed detection. Among these, YOLOv5 has gained widespread adoption thanks to its blend of speed, accuracy, and relatively user-friendly design. More specifically, YOLOv5s balances parameter efficiency with strong detection performance, making it highly attractive for real-time applications and further refinements in accuracy or speed.

Building on these strengths, various studies have explored YOLO-based enhancements to improve weed detection. For instance, Zhang et al. [14] proposed an optimized faster R-CNN for soybean field monitoring, and Zhang et al. [15] introduced EM-YOLOv4-Tiny for peanut field weed detection, surpassing the original YOLOv4-Tiny model through advanced mechanisms like attention modules and Soft-NMS. Pei et al. [16] implemented a novel YOLOv4-Tiny framework for maize row detection, whereas an improved YOLO-sesame attained a 96.16% mAP and 36.8 FPS in agricultural settings [17]. Meanwhile, YOLOv5n has proven faster in detecting cotton weeds, providing a suitable baseline for real-world needs [18]. Despite these accomplishments, challenges in handling diverse weed morphologies, varying sizes, and complex occlusions remain. To address these limitations, we propose YOLO-ACE—a precise and efficiency model built upon YOLOv5s, leveraging the latter’s favorable trade-off between performance and compactness. By introducing dual feature improvements and a decoupled detection head, YOLO-ACE enhances recognition accuracy without inflating the parameter count. Experimental validation demonstrates marked improvements in detecting smaller or occluded weeds under challenging field conditions, aligning with the pressing demand for reliable, high-speed weed detection in modern cotton production.

The main contributions of this research are highlighted below:

(1) The proposed model integrates dual-feature enhancement through the incorporation of a Context Augmentation Module (CAM) [19] and Selective Kernel Attention (SKAttention) [20]. CAM replaces the original Spatial Pyramid Pooling Fast (SPPF) [21], injecting contextual information into the feature pyramid network to strengthen the extraction of small-object features. Meanwhile, SKAttention, introduced after the C3 module [22] in the model’s neck, provides adaptive attention to features of varying target sizes, thus improving the model’s generalization ability.
(2) YOLO-ACE features an enhanced detection head that employs a decoupled detection head [23], separating the tasks of classification and bounding box regression. This design boosts detection performance and efficiency while minimizing task interference.
(3) The channel count M within SKAttention can be adjusted to meet different needs in real-world production environments. YOLO-ACE uses M = 4 by default. When M = 2, YOLO-ACE is designated as a lightweight model, termed YOLO-ACEs. The results show that YOLO-ACE excels in detecting small and shaded weeds in cotton weed detection, while maintaining high accuracy and excellent real-time detection speed.

The paper is organized as follows: Section 2 introduces the proposed model YOLO-ACE. Section 3 describes the dataset utilized and details the experimental validation of the proposed approach. Lastly, Section 4 concludes the study with a summary of key findings.

## 2. Methodology

### 2.1. YOLOv5

**Figure 1:**
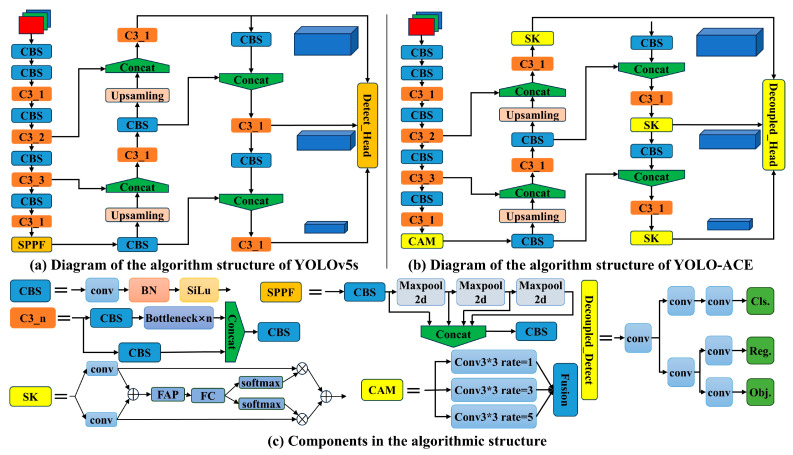
(**a**) Diagram of the algorithm structure of YOLOv5s; (**b**) diagram of the algorithm of YOLO-ACE; (**c**) components in the algorithmic structure.

YOLOv5 [22], a member of the You Only Look Once series [12,22,23,24,25,26,27], has been widely utilized in object detection due to its notable speed, accuracy, and user-friendly design. Numerous later models in the YOLO family can be viewed as extensions or refinements of YOLOv5. Depending on the scale of deployment, YOLOv5 provides five versions: YOLOv5n, YOLOv5s, YOLOv5m, YOLOv5l, and YOLOv5x. All versions share three core components: CSPDarkNet53 [26] as the backbone, a neck incorporating the Spatial Pyramid Pooling Fast (SPPF) module and Path Aggregation Network (PANet), and a YOLO head, as illustrated in Figure 1a.

Each version of YOLOv5 is designed to meet specific computational constraints, with differences primarily in model size, speed, and accuracy. As the light variant, YOLOv5s is particularly well suited for real-time weed detection in agricultural fields, where speed is crucial. Although previous studies have validated its potential for field-based weed detection [28], the model’s relatively low detection accuracy restricts its performance in more demanding tasks, such as weed identification in complex agricultural environments. To overcome this limitation, future approaches could explore the integration of enhanced modules aimed at improving detection performance without significantly compromising speed. While such enhancements may increase the model’s parameter count, optimizing the design of these modules could help reduce their impact on inference time. This approach holds promise for achieving a better balance between accuracy and speed, offering significant improvements in weed detection capabilities for real-time agricultural applications.

### 2.2. The Proposed Algorithm

Figure 1b illustrates an overview of the proposed YOLO-ACE model, built upon YOLOv5s and refined to address the challenges of cotton weed detection. In this framework, the original SPPF module is replaced by the Context Augmentation Module (CAM) to enhance multi-scale feature extraction, while the Selective Kernel Attention (SKAttention) mechanism is embedded within the neck to facilitate adaptive focus on diverse weed morphologies. Additionally, a decoupled head design replaces the standard YOLO head, aiming to improve both computational efficiency and detection accuracy. By adjusting the SKAttention channel parameter M, we cater to different resource constraints: setting M = 4 yields our primary YOLO-ACE configuration, reflecting the core principles of augmented feature extraction, contextual information enrichment, and heightened efficiency, whereas M = 2 produces YOLO-ACEs, a more compact variant retaining high performance. In the following sections, we detail how CAM, SKAttention, and the decoupled detection head individually contribute to the “ACE” strategy, collectively driving precise and efficient weed detection in cotton fields.

**Figure 2:**
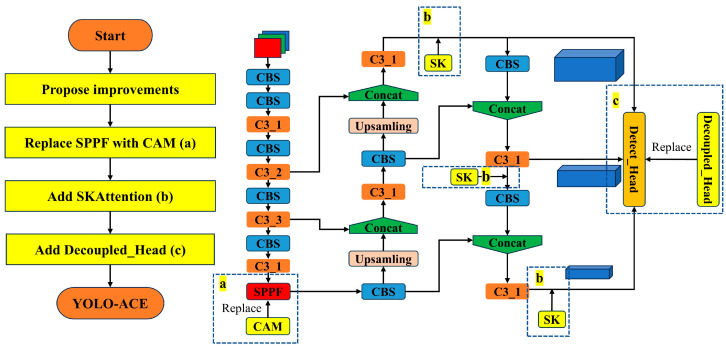
YOLO-ACE module integration flowchart.

Figure 2 presents a flowchart that visually demonstrates the integration process of the modules within the YOLO-ACE architecture. This diagram delineates the sequential operations, illustrating how the CAM replaces the SPPF module, how SKAttention is incorporated into the neck, and how the decoupled head substitutes the standard YOLO head.

### 2.3. Context Augmentation Module

As illustrated in Figure 1a, the Spatial Pyramid Pooling Fast (SPPF) module plays a pivotal role in bridging the backbone and the model’s neck. By employing convolution kernels of different sizes for pooling, it fuses multi-scale features and endows the network with robustness against scale variations. Nonetheless, SPPF exhibits limitations in accurately extracting small-scale targets, especially tiny weeds. This shortcoming motivated the introduction of the Context Augmentation Module (CAM), a component aligned with the “Augmented” aspect of YOLO-ACE, aiming to bolster the model’s capacity to capture fine-grained features.

**Figure 3:**
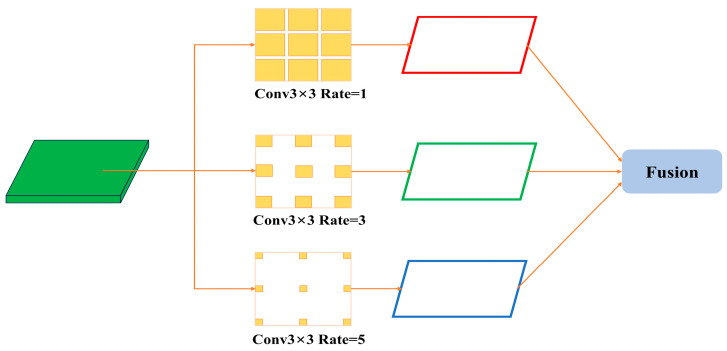
Network architecture diagram of Context Augmentation Module.

CAM inherits structural similarities from SPPF but is more purposefully designed to enhance weed detection performance. As depicted in Figure 3, CAM processes features via dilated convolutions with varied dilation rates (e.g., 1, 3, 5), enabling the network to glean richer spatial details across multiple scales. Through subsequent fusion operations, CAM aggregates these receptive fields into a unified representation, thus injecting additional contextual cues. In this manner, CAM effectively addresses the detection of small or occluded weed targets, improving the model’s overall accuracy in challenging field environments.

**Figure 4:**
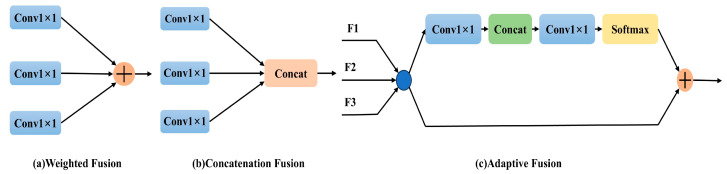
Fusion methods of CAM: (**a**) and (**b**) show direct feature map integration via weighting and concatenation, respectively, while (**c**) employs an adaptive fusion—combining convolution, splicing, and softmax—to merge information from three channels.

In further detail, CAM offers three distinct feature fusion strategies—labeled (a), (b), and (c)—shown in Figure 4. Methods (a) and (b) employ weighting and concatenation, respectively, for direct integration of feature maps across spatial and channel dimensions. In contrast, method (c) introduces an adaptive fusion scheme by combining convolution, splicing, and softmax operations. This adaptive scheme dynamically merges contextual information from three separate input channels, producing a refined output that heightens sensitivity to subtle target features. A comprehensive evaluation of these strategies is provided in the experimental section, underscoring CAM’s role in augmenting feature extraction, a core tenet of the “A” in YOLO-ACE.

### 2.4. Selective Kernel Attention

Field environments often present weeds with varied morphologies and sizes, which can cause critical target features to be overlooked during feature extraction. To address this challenge, the Selective Kernel Attention (SKAttention) mechanism is integrated into the network’s neck, corresponding to the “Contextual” component of YOLO-ACE. By adaptively redistributing attention, SKAttention focuses computational resources on significant weed features, mitigating information loss and boosting detection accuracy in diverse field settings.

**Figure 5:**
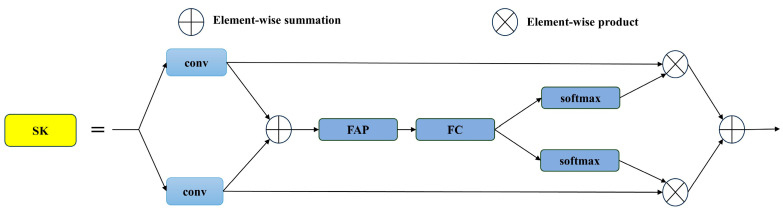
Selective kernel attention.

Figure 5 illustrates SKAttention’s structure, which leverages multiple convolutional kernel sizes in a non-linear fashion, enabling dynamic adjustment of the receptive field. This mechanism comprises three principal operators—Split, Fuse, and Select. The “Split” phase spawns multiple branches, each adopting a different kernel size to extract features from various receptive field dimensions. The “Fuse” operator then amalgamates these multi-branch features into a comprehensive global representation for weight computation. Lastly, the “Select” operator merges the per-branch feature maps in accordance with the learned weights, preserving the most pertinent contextual cues. Through this multi-stage process, SKAttention enriches the network’s representation of weed targets, fulfilling the “Contextual” requirement of YOLO-ACE by adapting its focus to the intricacies of real-world cotton weed detection scenarios.

### 2.5. Decoupled Detection Head

Prior research confirms that incorporating a decoupled detection head into the YOLOv3 architecture yielded a 1.1 percentage point improvement in precision, highlighting its structural efficacy. This approach has been further validated in the YOLOX [29] project and subsequently refined in models such as YOLOv6. The decoupled head treats classification and localization as separate tasks, each handled by an individual branch, thereby reducing interference between task-specific features [30].

In YOLO-ACE, this decoupled design underpins the “Efficiency” dimension, optimizing the computational process for real-time weed detection without sacrificing accuracy. By allocating distinct branches to classification and bounding box regression, the model more efficiently handles the heterogeneous feature demands of each task. In practice, this structural refinement lowers inference latency while retaining robust performance, striking a balance between computational constraints and detection precision crucial for large-scale agricultural applications.

Moreover, this study embraces the “Hybrid Channels” concept from YOLOv6 to mitigate the additional overhead of conventional 3 × 3 convolutions in decoupled heads. The streamlined head effectively narrows the gap between high accuracy and real-time processing—an essential factor in field-deployable weed detection solutions. Thus, the decoupled detection head not only heightens the model’s overall effectiveness but also symbolizes the “E” (efficiency) in YOLO-ACE, ensuring that the model sustains the throughput needed for practical usage scenarios.

## 3. Results and Analysis

### 3.1. Dataset

**Figure 6:**
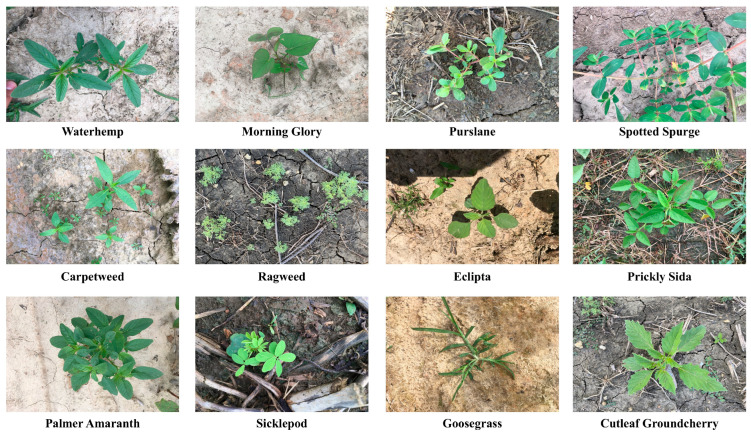
Examples of the 12 categories of weeds in CottonWeedDet12.

A comprehensive cotton weed dataset, featuring 15 weed classes from cotton fields in the United States and comprising a total of 5187 images, was recently introduced [31]. Thirty-five cutting-edge deep learning models were assessed through transfer learning and applied for weed classification, achieving an accuracy of over 95%. This dataset was confined to the classification task alone, owing to the absence of bounding box annotations for weed boundaries. Dang et al. introduced a novel dataset named CottonWeedDet12, comprising 5648 images across 12 weed classes, complete with bounding box annotations. Upon evaluating 25 YOLO object detectors using this dataset, they discovered that YOLOv4 attained the highest detection accuracy, while YOLOv5s offered the optimal balance of detection accuracy and speed [28]. Given that this dataset not only encompasses data on various weed types and a public collection of bounding boxes but also serves as a benchmark for weed detection across the YOLO model series, selecting this dataset is more pertinent. On one hand, it circumvents the necessity of collecting and labeling the data, allowing for greater focus on enhancing model performance; on the other hand, CottonWeeddet12, as a publicly available dataset for weed detection, facilitates more comprehensive testing of the model’s capabilities and contributes significantly to future weed detection and identification efforts. Consequently, this dataset has been selected for use in this study to achieve efficient cotton weed detection. Figure 6 presents an example of the 12 weed classes featured in this dataset. Most images in CottonWeedDet12 are high-resolution, typically exceeding 1000 pixels in at least one dimension—for instance, some images are around 3024 × 2032. We split the dataset into training, validation, and testing sets with a 7:2:1 ratio, ensuring a balanced and comprehensive evaluation of our model. In addition, we employed multiple data augmentation strategies—such as mosaic, random flipping, and HSV (hue, saturation, value) adjustments—to enhance the robustness and generalizability of our model.

### 3.2. Experimental Environment and Evaluating Indicators

The experiment was conducted on a Linux system equipped with a 15 vCPU Intel(R) Xeon(R) Platinum 8358P CPU at 2.6 GHz, 80 GB of RAM, an Nvidia RTX A5000 graphics card, CUDA version 11.8, CUDNN version 8.0, Python 3.8, and the PyTorch 2.0. The learning rate was initially configured to 0.01, while the momentum was set to 0.937, weight decay coefficient at 0.0005, image input size at 640 × 640 pixels, batch size at 16, and the training was conducted over 300 epochs.

This study employs the fundamental evaluation metrics from the field of object detection to undertake a comprehensive evaluation and analysis of the proposed improved model. Specifically, this evaluation encompasses precision (P), recall (R), and mean average precision (mAP), with particular emphasis on performance at a singular threshold of 0.5 (mAP@0.5) in comparison to a spectrum of thresholds ranging from 0.5 to 0.95 (mAP@0.5:0.95). Additionally, Frames Per Second (FPS) and parameter count are also incorporated as key metrics to assess the model’s computational efficiency and scalability. The combined use of these metrics aims to thoroughly evaluate the model’s precision and stability in detection tasks, providing a scientific and quantitative basis for judging the model’s performance in practical application environments.

(1) $$Precision(P)=\frac{TP}{TP+FP}$$

(2) $$Recall(R)=\frac{TP}{TP+FN}$$

(3) $$AP=\int_{0}^{1}P\left(R\right)dR$$

(4) $$mAP=\frac{1}{n}\sum_{i=1}^{n}AP(i)$$

Within the assessment framework of this research, the true positives (*TP*) metric indicates the count of samples correctly identified as positive, whereas the false positives (*FP*) denote instances incorrectly labeled as positive but were in fact neXgative. Conversely, false negatives (*FN*) refer to instances that are incorrectly predicted as negative when they are actually positive. In the mathematical representation, the symbol i refers to a specific species category, while AP(i) represents the average precision corresponding to that category and *n* represents the total number of categories. In addition, the “0.5” in mAP@0.5 refers to the threshold of Intersection over Union (IoU) set at 0.5. IoU is an evaluation metric to quantify the overlap between the predicted bounding box and the actual bounding box, which is calculated as the ratio of the intersection area and the union area of two bounding boxes. Predictions are considered accurate only if the IoU meets or exceeds the 0.5 criterion. mAP@0.5:0.95 involves the average of the mean precision computed for a series of IoU thresholds ranging from 0.5 to 0.95 in 0.05 increments, thus providing a comprehensive assessment of model performance at multiple levels of accuracy.

### 3.3. Comparison Between the Improved Model and YOLOv5s

To assess the effectiveness of the proposed YOLO-ACE framework, we conducted experiments under identical conditions and hyperparameters on both YOLOv5s and our enhanced models. The comparative results are reported in Table 1, revealing clear improvements in precision, recall, and both mAP@0.5 and mAP@0.5:0.95. Notably, mAP@0.5 and mAP@0.5:0.95 reached impressive peaks of 95.3% and 89.5%, respectively, substantiating the benefits of our augmented contextual efficiency design. These outcomes indicate a pronounced increase in weed detection accuracy, a critical factor in ensuring reliable performance in actual field applications. A more detailed analysis of these metrics in subsequent sections further underscores YOLO-ACE’s advanced detection capabilities. While the added modules in YOLO-ACE are primarily designed for efficiency, we recognize that even minor structural changes can lead to an overall increase in parameter count as the network deepens. Nevertheless, our specially tailored architecture demonstrates superior experimental results compared to YOLOv5x, which features a similar structure and parameter scale. Moreover, FPS calculations confirm that our model effectively meets real-time detection requirements, achieving an excellent balance between speed and accuracy.

**Figure 7:**
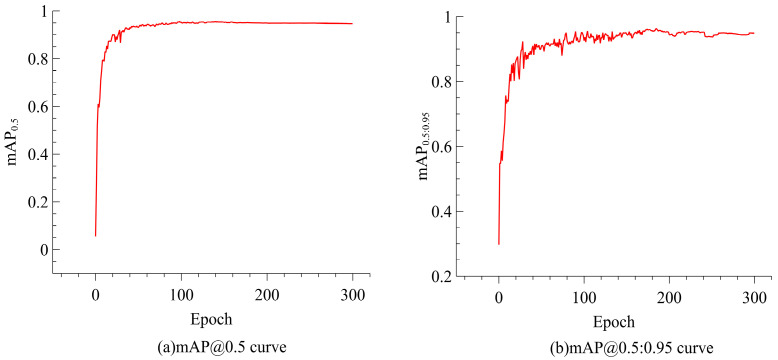
Convergence curves of YOLO-ACE: (**a**) mAP at IoU = 0.5; (**b**) mAP across IoU thresholds from 0.5 to 0.95.

Figure 7 depicts the model’s mAP@0.5 and mAP@0.5:0.95 curves, illustrating a consistent upward trend in accuracy throughout the training process. As the IoU threshold intensifies (from 0.5 to 0.95), YOLO-ACE continues to perform robustly, evidencing its adaptability to stringent detection requirements.

**Figure 8:**
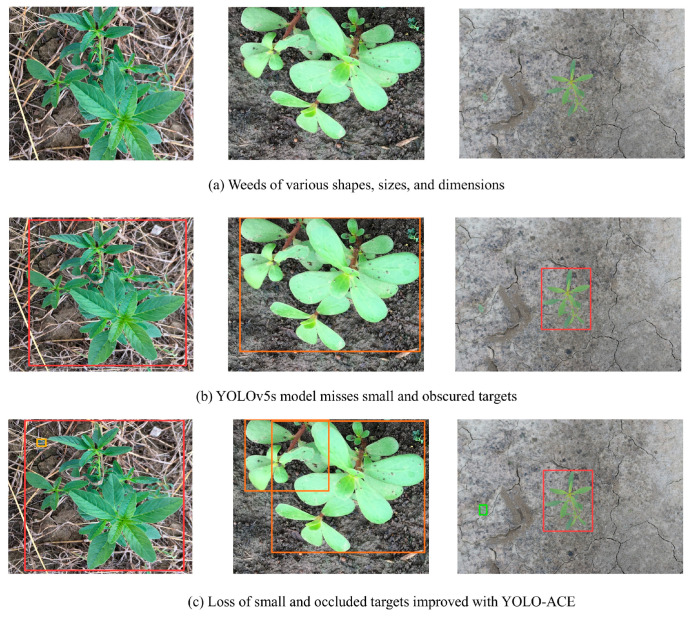
Detection Comparative Analysis of Cotton Weed Detection under Challenging Conditions: (**a**) Weeds exhibiting diverse shapes, sizes, and dimensions; (**b**) YOLOv5s failing to detect small and occluded targets; (**c**) YOLO-ACE demonstrating enhanced detection of small and occluded targets.

Additionally, real-world scenarios (Figure 8) demonstrate the model’s competence in handling occluded and diminutive weed targets—circumstances in which YOLOv5s often struggles. In Figure 8b, YOLOv5s misdetects heavily occluded weeds, whereas YOLO-ACE (Figure 8c) accurately identifies these challenging targets (highlighted in yellow), confirming its superior efficacy for precision cotton weed detection. These findings validate the overarching goal of YOLO-ACE: to deliver an efficient, high-accuracy solution for the complexities of practical agricultural environments.

### 3.4. Comparative Performance Analysis of Improved Models on CottonWeedDet12

To further validate the capabilities of our enhanced framework, we benchmarked YOLO-ACE against a high-performing reference model, selecting the data that displayed the strongest metrics overall. The results, presented in Table 2, reveal that despite having comparatively fewer parameters, our model maintains top-tier accuracy and mAP@0.5, achieving 96.1% and 95.3%, respectively. Moreover, its mAP@0.5:0.95 falls just 0.2 percentage points shy of the leading benchmark. Additionally, YOLO-ACEs—our most lightweight variant—also demonstrates commendable performance, lagging only 0.3 points behind the highest mAP@0.5 metric. This underscores YOLO-ACE’s high potential for real-time weed detection, seamlessly blending accuracy with a reduced parameter footprint. In terms of parameter count, YOLO-ACE requires fewer than half the parameters of Scaled-yolov4-p6 and under one-fifth of Scaled-yolov4-p7—two models known for top-tier recall and mAP@0.5:0.95. This substantial reduction highlights the effectiveness of our “Augmented Contextual Efficiency” design philosophy in meeting practical agricultural needs. The option to select either YOLO-ACE or YOLO-ACE-S allows users to adapt to varying resource constraints and environmental conditions, aligning with the overarching aim of delivering a precise, efficiency, and adaptable weed detection model for real-world cotton fields. Furthermore, we have further assessed the FPS and parameter scale of our models, revealing that YOLO-ACE achieves 77 FPS and YOLO-ACEs reaches 92 FPS, both featuring parameter counts lower than mainstream models such as YOLOv3, YOLOv4, and YOLOv5x. Although our parameter count is slightly higher than YOLOv6s, our FPS meets the needs for real-time detection, and our average precision surpasses that of YOLOv6s, achieving a better balance between speed and accuracy.

### 3.5. Ablation Experiment

In our ablation study, YOLOv5s served as the foundational model to assess the impact of the Context Augmentation Module (CAM), SKAttention (M = 4), and the decoupled detection head. As shown in Table 3, the baseline YOLOv5s achieved 93.86% precision, 89.22% recall, 94.02% mAP@0.5, and 86.6% mAP@0.5:0.95.

Integrating CAM elevated the model’s mAP@0.5:0.95 to 87.4%, with precision and recall holding steady, suggesting that CAM effectively enhances small-object detection. Adding the SKAttention mechanism led to a modest drop in recall (to 84.1%), yet increased mAP@0.5 and mAP@0.5:0.95 to 94.6% and 87.2%, respectively, reflecting its capacity to refine feature extraction. Incorporating a decoupled detection head further boosted precision to 94.2%, with mAP@0.5 and mAP@0.5:0.95 reaching 94.4% and 87.1%, although recall dipped slightly to 89.1%. When combining these modules, synergistic effects emerged. Merging CAM and the decoupled detection head improved all metrics, with precision at 94.3% and mAP@0.5:0.95 at 87.7%. Similarly, CAM plus SKAttention raised precision to 94.4%, recall to 89.3%, mAP@0.5 to 94.8%, and mAP@0.5:0.95 to 89.0%. Meanwhile, the integration of SKAttention and the decoupled head produced a notable increase across every metric, culminating in precision of 94.1%, recall of 90.0%, mAP@0.5 of 94.7%, and mAP@0.5:0.95 of 89.1%. Finally, employing all three modules simultaneously yielded peak scores in every category: 96.1% precision, 89.6% recall, 95.3% mAP@0.5, and 89.5% mAP@0.5:0.95. This remarkable outcome reinforces the central premise of YOLO-ACE—that layered improvements in feature augmentation, contextual awareness, and efficiency can significantly enhance weed detection performance. Each individual component contributes to this boost, and their collective integration magnifies it further, underscoring the robustness and adaptability of YOLO-ACE for precision cotton weed detection.

**Figure 9:**
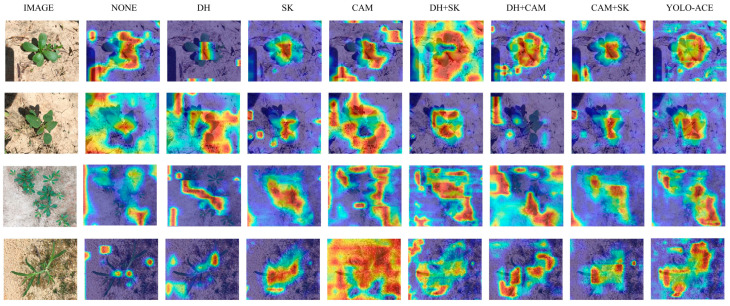
Comparative heatmap visualizations of YOLOv5 variants with module integrations—none, decoupled head (DH), SKAttention (SK), Context Augmentation Module (CAM), and Full Integration (YOLO-ACE).

To further assess the interpretability and localized focus of these enhancements, we utilized Grad-CAM++ [32] to generate heatmaps highlighting the regions most influential in the model’s decision-making process. Figure 9 shows that incorporating the CAM, SKAttention, and the decoupled detection head leads to more precise emphasis on weed regions. Notably, the full combination of all three modules demonstrates the highest concentration on target weeds and the least background confusion, thereby offering a clearer insight into how YOLO-ACE makes detection decisions. This comprehensive visualization indicates that each module not only contributes to performance gains but also improves model interpretability in real-world cotton field scenarios.

### 3.6. Explored the Performance of Three Feature Fusion Strategies in the CAM

This section explores three distinct feature fusion strategies in the Context Augmentation Module (CAM)—adaptive, concatenated, and weighted—to determine their influence on the YOLO-ACE framework. Table 4 summarizes the empirical findings. For YOLO-ACE-S, the weighted fusion strategy yields a peak accuracy of 95.5%, highlighting its effectiveness in bolstering detection tasks. Meanwhile, concatenation excels in recall and average accuracy (mAP@0.5 and mAP@0.5:0.95), reaching 90.9%, 95.0%, and 88.6%, respectively. In the YOLO-ACE model configuration, the adaptive fusion approach secures the highest recall at 89.8%, whereas the concatenation method achieves top precision and notable mAP scores of 95.3.% (mAP@0.5) and 89.5% (mAP@0.5:0.95).

Although concatenation offers the most balanced overall performance in these tests, both adaptive and weighted strategies also show strong results. This outcome underscores the versatility of different fusion approaches under the “Augmented Contextual Efficiency” paradigm and suggests that each can be fine-tuned for various network architectures or specific agricultural scenarios. Ultimately, these findings reaffirm CAM’s essential role in enabling more nuanced feature extraction, aligning well with the core objective of YOLO-ACE to deliver precise and efficient weed detection in real-world cotton fields.

### 3.7. Evaluation of Our Model on the CropWeed Dataset

To further validate our model in broader contexts, we assessed it on the CropWeed dataset. The CropWeed dataset consists of 1300 images of sesame crops and different types of weeds, with each image labeled. Each image is a 512 × 512 color image. Maintaining consistent experimental parameters, we evaluated YOLO-ACE on this dataset, and the results are presented in Table 5. YOLO-ACE achieves an accuracy of 84.3% in mAP@0.5 and 57.0% in mAP@0.5:0.95, with only 50 million parameters—surpassing all benchmark models in mAP@0.5:0.95. Notably, it surpasses YOLOv7x (52.5% mAP@0.5:0.95) by 4.5 percentage points, while also outperforming it in parameter efficiency and inference speed. Moreover, YOLO-ACEs attains a high mAP@0.5:0.95 among the compared methods, maintaining high mAP@0.5 scores with even fewer parameters. This reduction in parameters proves particularly advantageous for resource-limited devices and more complex weed environments, emphasizing the adaptability inherent in the “Augmented Contextual Efficiency” strategy.

**Figure 10:**
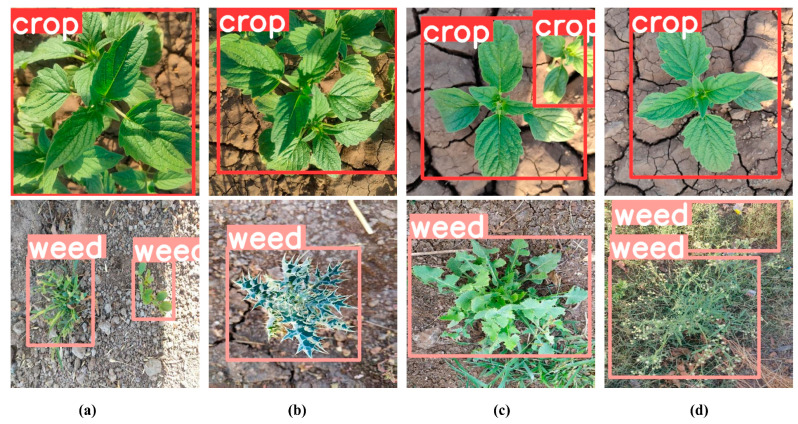
Robust Weed Detection under Variable Conditions: (**a**–**d**) present detection outcomes under diverse lighting conditions and viewing angles.

Additionally, we discussed the robustness of the model under different lighting conditions and viewpoints, demonstrating its ability to effectively detect weeds under these variations. As shown in Figure 10, we present the detection results under varying lighting conditions and viewpoints, where both sesame crops and weeds are effectively detected. Overall, these experiments highlight YOLO-ACE’s significant potential in field weed detection and emphasize its practical advantages for large-scale agricultural applications.

### 3.8. Challenges and Limitations in YOLO-ACE’s Weed Detection Performance

**Figure 11:**
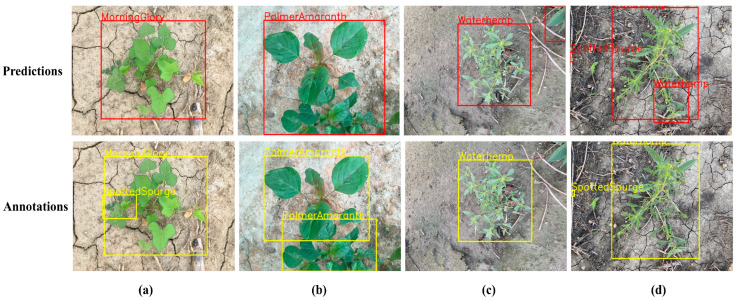
Analysis of YOLO-ACE Detection Failures: (**a**,**b**) reveal that severe occlusion or overlap can lead to missed weed detections due to inherent ambiguities. (**c**,**d**) show that enhanced feature extraction may misclassify subtle plant features as weeds, resulting in false positives.

Despite the overall success of YOLO-ACE, certain edge cases remain challenging. As illustrated in Figure 11, the model encounters difficulties in specific scenarios. For example, in severely occluded scenes (Figure 11a) or heavily overlapped regions (Figure 11b), YOLO-ACE occasionally struggles to detect the weed targets accurately, resulting in missed detections. This issue arises from the inherent ambiguity in such situations—ambiguities that even human observers may find difficult to resolve without careful inspection—indicating a need for more sophisticated attention mechanisms. On the other hand, Figure 11c,d reveal instances where subtle plant features are incorrectly classified as weeds, likely due to the model’s enhanced feature extraction capabilities. While reinforcing feature extraction improves detection accuracy in many cases, it can also increase the model’s sensitivity to complex backgrounds, leading to false positives. These observations emphasize the need for further investigation into advanced attention modules or contextual cues in future work to mitigate both false negatives and false positives.

## 4. Conclusions

In this study, we introduced YOLO-ACE—a high-precision extension of YOLOv5s—along with a more compact variant, YOLO-ACEs, both tailored to cotton weed detection. By replacing the original Spatial Pyramid Pooling with a Context Augmentation Module (CAM), we enriched multi-scale feature extraction; meanwhile, the SKAttention mechanism in the neck enhanced recognition of various weed sizes and shapes. A decoupled detection head further improved accuracy and efficiency, underscoring the “Augmented Contextual Efficiency” principle at the heart of YOLO-ACE. Experimental evaluations on CottonWeedDet12 demonstrated that YOLO-ACE attained top precision and mAP@0.5 while consuming fewer than half—or even one-fifth—of the parameters used by Scaled-YOLOv4-p7, without sacrificing mAP@0.5:0.95 or recall. YOLO-ACEs, albeit slightly less precise, halved the parameter count, making it particularly suitable for real-time tasks in resource-constrained scenarios. Tests on the CropWeed dataset confirmed these findings, demonstrating that our model can be extended to other types of weeds, with YOLO-ACE exhibiting robustness across different environments. Overall, the YOLO-ACE framework strikes a favorable balance between speed, accuracy, and parameter efficiency, meeting the stringent requirements of modern cotton weed management. Moving forward, we aim to refine the architecture’s accuracy and efficiency even further, broadening its applicability in large-scale agricultural environments and advancing precision weed detection.

## Figures and Tables

**Table 1:** Comparison between the improved model and YOLOv5.

Models	Precision	Recall	mAP@0.5	mAP@0.5:0.95	FPS	Parameters (Million)
YOLOv5s	93.9	89.2	94.0	86.6	112	7
YOLOv5x	94.9	88.9	94.8	88.9	40	87
YOLO-ACEs	94.4	90.9	95.0	88.6	92	25
YOLO-ACE	96.1	89.6	95.3	89.5	77	50

**Table 2:** Performance comparison of YOLO models.

Models	Precision	Recall	mAP@0.5	mAP@0.5:0.95	FPS	Parameters (Million)
YOLOv3	94.0	90.6	93.6	87.0	57	62
YOLOv3-SPP	94.8	89.9	94.3	87.5	53	63
YOLOv4	81.3	95.0	95.2	89.5	65	64
Scaled-yolov4-p6	85.1	94.7	95.1	89.7	29	128
Scaled-yolov4-p7	81.3	94.9	95.2	89.4	16	287
YOLOv5x	94.9	88.9	94.8	88.9	40	87
YOLOv6s	93.9	88.6	93.5	88.2	107	19
YOLOv7x	93.8	91.2	94.7	89.3	47	71
YOLO-ACEs	94.4	90.9	95.0	88.6	92	25
YOLO-ACE	96.1	89.6	95.3	89.5	77	50

**Table 3:** Ablation experiment results.

Models	CAM	SKAttention	Decoupled Head	Precision	Recall	mAP@0.5	mAP@0.5:0.95
YOLOv5s				93.8	89.2	94.0	86.6
	**✓**			93.9	89.2	94.2	87.4
		**✓**		94.1	84.1	94.6	87.2
			**✓**	94.2	89.1	94.4	87.1
	**✓**	**✓**		94.3	89.3	94.3	87.7
	**✓**		**✓**	94.4	89.3	94.8	89.0
		**✓**	**✓**	94.1	90.0	94.7	89.1
	**✓**	**✓**	**✓**	96.1	89.6	95.3	89.5

**Table 4:** Performance of three feature fusion strategies in the CAM.

Models	Weight	Adaptive	Concatenation	Precision	Recall	mAP@0.5	mAP@0.5:0.95
YOLO-ACEs	**✓**			95.5	89.3	94.9	88.4
		**✓**		94.6	88.4	94.7	88.6
			**✓**	94.4	90.9	95.0	88.6
YOLO-ACE	**✓**			94.8	89.7	95.2	89.1
		**✓**		94.7	89.8	94.9	89.2
			**✓**	96.1	89.6	95.3	89.5

**Table 5:** Comparison of object detection models on CropWeed dataset.

Models	Parameters (Million)	FPS	mAP@0.5	mAP@0.5:0.95
YOLOv3	62	57	81.8	51.9
YOLOv4	64	65	82.3	56.9
YOLOv5x	87	40	82.2	53.5
YOLOv6s	19	107	83.6	55.0
YOLOv7x	71	47	82.6	52.5
YOLO-ACEs	25	92	83.6	56.7
YOLO-ACE	50	77	84.3	57.0

## Data Availability

This article utilizes public datasets CottonWeeddet12 (https://zenodo.org/records/7535814 accessed on 13 September 2023) and CropWeed (https://www.kaggle.com/datasets/ravirajsinh45/crop-and-weed-detection-data-with-bounding-boxes accessed on 18 September 2024).
